# Choroid plexus carcinoma in an adolescent male: a case report

**DOI:** 10.1186/s13256-021-02801-w

**Published:** 2021-04-21

**Authors:** Patel Zeeshan Jameel, Ashish Varma, Pooja Kumari, Keta Vagha, Jayant Vagha, Sachin Damke

**Affiliations:** Department of Paediatrics, Jawaharlal Nehru Medical College, Sawangi (Meghe), Wardha, Maharashtra 442001 India

**Keywords:** Choroid plexus carcinoma, Brain tumor, Gross total resection

## Abstract

**Introduction/background:**

Although central nervous system tumors are the most common etiology of malignancies in the pediatric age group, choroid plexus carcinomas are rare, with an annual incidence rate of 0.10 per 100,000 children.

**Case presentation:**

We report the case of an adolescent male belonging to central India who had presented with a history of persistent headache, projectile vomiting, neck stiffness, and an episode of generalized tonic-clonic seizure. Neurological examination was suggestive of a space-occupying lesion. Further neuroimaging was suggestive of a large left-sided choroid plexus carcinoma, later confirmed on pathological examination. Gross total resection was achieved and followed by radiation therapy. His recovery was satisfactory without any major events despite suffering from such a malignancy with a poor prognosis.

**Conclusion:**

In the absence of a global consensus on choroid plexus carcinoma management, our patient underwent a successful gross total resection and received postoperative radiotherapy. He made a satisfactory recovery with a further plan to review with gadolinium-enhanced neuroimaging at a later date. We conclude that, when possible, achieving gross total resection is of utmost importance.

## Introduction

Central nervous system (CNS) tumors are the most common etiology of cancer in the pediatric age group (0–19 years) with an incidence rate of 5.57 per 100,000 population [1]. Among them, choroid plexus tumors (CPTs) have an average annual age-adjusted incidence rate of 0.10 per 100,000 children, being most common in the age group 0–4 years [1]. Overall, CNS tumors may have grave consequences, with a mortality rate of 0.65 per 100,000 children.1 As per the World Health Organization (WHO) classification, CPTs are further subclassified into choroid plexus papilloma (CPP; WHO grade I), atypical choroid plexus papilloma (WHO grade II), and choroid plexus carcinoma (CPC; WHO grade III) [2].

CPC is an exceptionally rare intracranial neoplasm characterized histologically by friable papillary or cauliflower-like appearance, increased mitotic figures, pleomorphic nuclei, and necrosis [3]. CPC was found to be more common among males and significantly associated with younger age (median age 1 year) at diagnosis in comparison with CPP [4]. The clinical presentation is determined by the age of onset, as well as location and size of the tumor. Current management strategies, although not standardized, include gross total resection of the tumor followed by chemotherapy and radiation therapy (RT).

Here, we describe a rare case of pediatric CPC in the lateral ventricle of an adolescent male and briefly review the current management strategies.

## Case presentation

A 14-year-old adolescent male belonging to central India presented to our tertiary care health facility in central India with a history of persistent headache for 2 weeks, projectile vomiting for 5 days, neck stiffness for 5 days, and a single episode of generalized tonic-clonic seizure (GTCS) lasting for 10 minutes. Papilledema was present. Systemic examination showed right-sided hemiparesis (power 2/5) with exaggerated deep tendon reflexes and a positive Babinski sign suggestive of an intracranial space-occupying lesion (SOL).

**Fig. 1:**
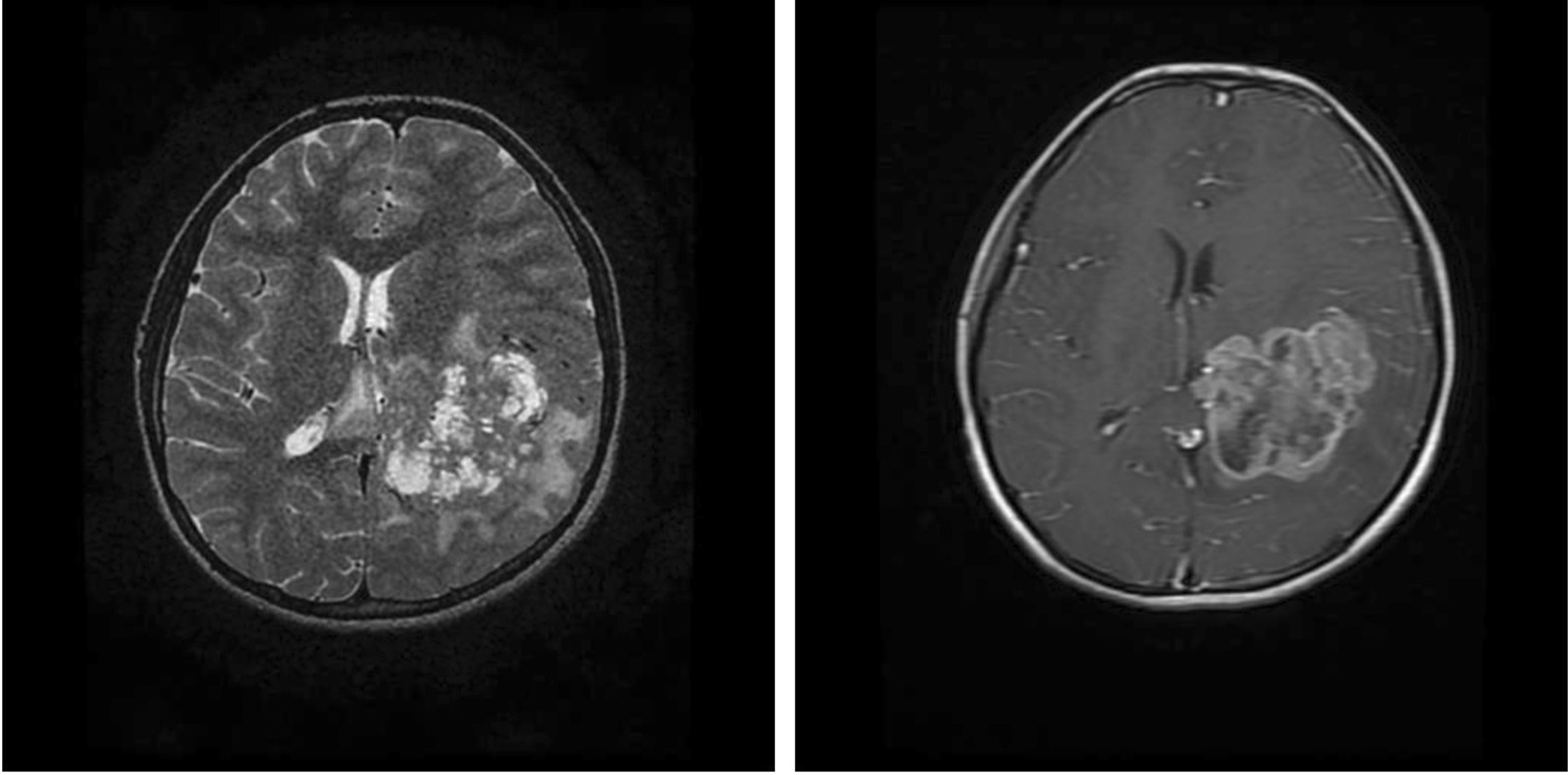
**a** T2 weighted image (T2WI) showing heterogeneous space occupying lesion (SOL) with necrotic areas with epicenter in left trigone of lateral ventricle infiltrating into adjacent white matter with surrounding vasogenic edema. (**b**) Postcontrast T1WI showing heterogeneous enhancement with multiple nonenhancing necrotic areas

After initial stabilization, brain magnetic resonance imaging (MRI) revealed a heterogeneously enhancing mass (6.2 × 5.2 × 4 cm), located in the left lateral ventricle extending diffusely into the ipsilateral parietooccipital region with perilesional edema with a midline shift of 6 mm (Fig. 1). There were no signs of spinal cord involvement. Magnetic resonance (MR) spectroscopy showed reduced *N*-acetyl-aspartate (NAA) and increased choline, with a lactate peak suggestive of a malignant mass.

**Fig. 2:**
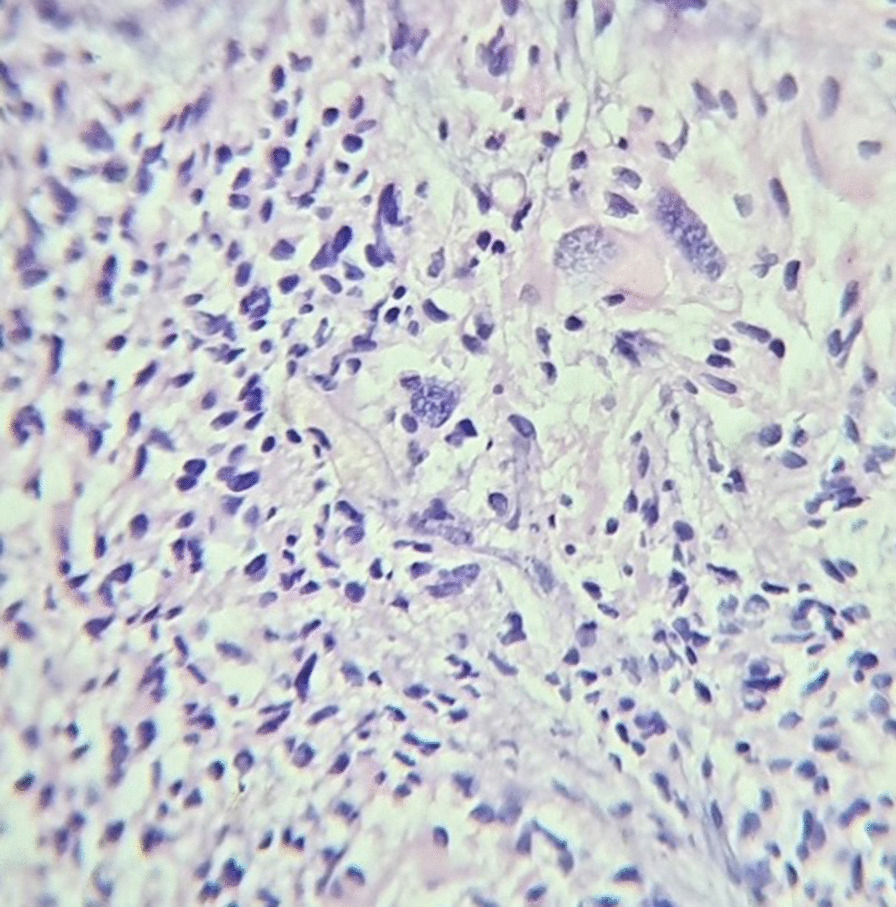
Histological examination showing crowded and branched papillae merging to form sheets of tumor cells disrupted by areas of necrosis with tumor cells exhibiting nuclear pleomorphism with readily identifiable mitoses

Gross total excision of the tumor was achieved with coagulation of the choroid plexus. Intraoperative findings revealed a soft, cystic, necrotic, and highly vascular tumor with intraventricular extension. Histopathological examination revealed crowded and branched papillae merging to form sheets of tumor cells disrupted by areas of necrosis with tumor cells exhibiting nuclear pleomorphism (multilobulated, multinucleated hyperchromatic nuclei). Mitoses were readily identified (15–20/10 hpf). Surrounding brain parenchyma infiltration was also noted (Fig. 2). Immunohistochemistry showed tumor cells expressing cytokeratin (focal) and P53 (diffuse) but did not express synaptophysin, glial fibrillary acidic protein (GFAP), or epithelial membrane antigen (EMA). Also, MIB-1 nuclear labeling was increased markedly, with the index being approximately 40% in areas of highest proliferative activity. Hence, a diagnosis of choroid plexus carcinoma (CPC) was rendered.

**Fig. 3:**
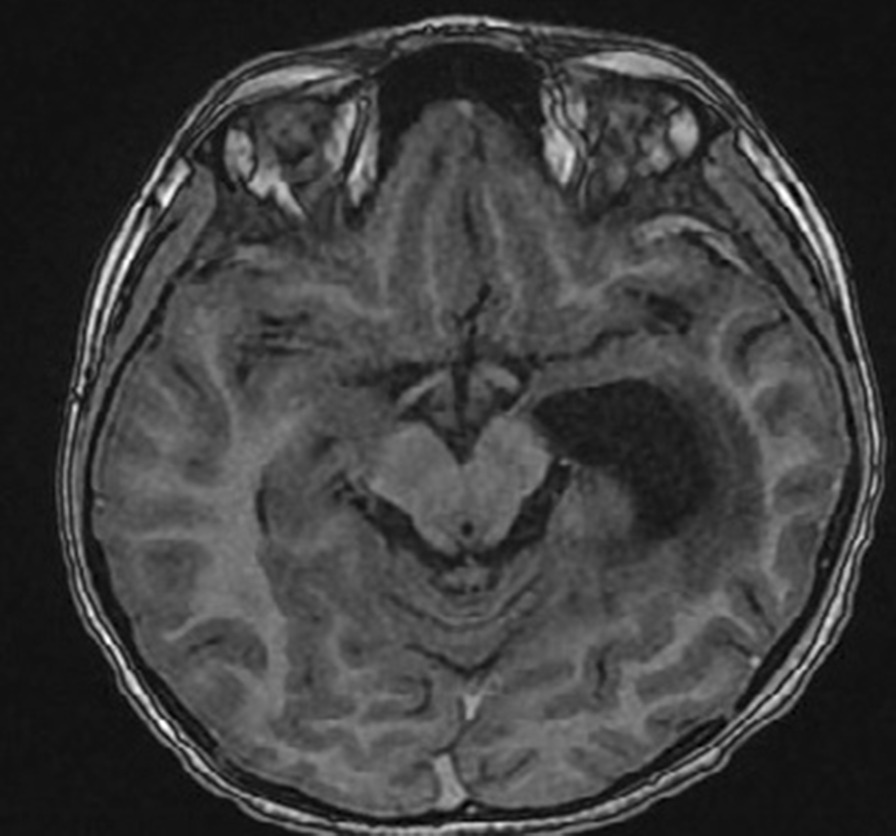
Postsurgery T1WI showing a resection cavity in the left temporoparietal lobes with no signs of any residual neoplastic tissue

The patient’s postoperative recovery was satisfactory with improved power (5/5 on the right side) and resolution of all symptoms. The patient was planned for adjuvant radiotherapy. He received 59 Gy of cranial radiation in 33 fractions using intensity-modulated radiation therapy (IMRT). Follow-up imaging after 3 months showed no residual neoplastic lesion with no metastatic lesion elsewhere on CNS imaging (Fig. 3). The further management plan includes close follow-up for the next 6 months with neuroimaging again 1 year postsurgery.

## Discussion

Choroid plexus carcinoma (CPC) is a rare cause of a hemispheric cerebral tumor arising from lateral ventricles in children. The possible differential diagnosis for such a hemispheric brain tumor includes choroid plexus papilloma (CPP), ependymoma, atypical teratoid rhabdoid tumor, glioma, astrocytoma, and primitive neuroectodermal tumor (PNET). Radiopathological correlation with tissue immunohistochemistry is of essence in differentiating and establishing a confirmatory diagnosis.

The extremely low incidence of CPC in children has been a major obstacle in the development of standardized clinical trials with the therapeutic options being based upon expert opinion and case studies. Various management strategies include surgery, chemotherapy, radiotherapy, and autologous hematopoietic cell rescue.

Surgery remains to be the cornerstone of management, and the completeness of resection influences patient survival. Achieving gross total resection (GTR) is the most decisive factor for a patient’s long-term survival and prognosis. Various studies have determined that patients who have undergone GTR have significantly better survival rates [5, 6]. Furthermore, Mallick *et al*. [7] observed that progression-free survival was significantly higher for patients with GTR in comparison with subtotal resection (60 months versus 11 months) after eliminating the impact of adjuvant therapy. In addition, the extent of surgical resection remained a significant factor affecting survival [7]. Despite the merits of GTR, it is often difficult to achieve complete resection and it has increased morbidity. The large size, high vascularity, diffuse infiltrative nature, and excessive friability of CPC present a formidable challenge for complete resection. In the pediatric population, blood loss may be life threatening as the entire circulating blood volume may be lost during the resection of these vascular tumors. The surgical approach planned should allow good visual access to vascular supply and maximal exposure of the tumor mass. An effective intraoperative surgical strategy is to identify and ligate the feeding choroidal vessel, thus facilitating the en bloc tumor mass removal. In patients with large tumors where the tumor is resected in parts, gentle coagulation of the fronds of the tumor allows for manipulation without excessive bleeding and may reduce the tumor size. If complete resection is not achieved for any reason, a second-look surgery may achieve an eventual GTR, or this surgery may be preceded by administration of chemotherapy, which will to reduce intraoperative bleeding and tumor size, thus allowing for a complete subsequent resection rather than an incomplete resection [8–10]. In our patient, a successful GTR was performed via a transcortical approach.

A global consensus on neoadjuvant chemotherapy and regimens is lacking and is yet to be standardized. The following drugs are used in the treatment: carboplatin, etoposide, cyclophosphamide, high-dose methotrexate, and vinca alkaloids [11]. All meta-analyses have focused primarily on the benefits of using chemotherapy, without a focus on a particular regimen or agent. Using multivariate Cox regression survival analysis, Sun *et al*. [12] confirmed a better prognosis and a significantly better cumulative overall survival in children with CPC receiving chemotherapy alone. However, the implementation of combined chemoradiotherapy had better overall survival than chemotherapy alone [12]. Besides, among patients with incomplete resection of CPC, chemotherapy was found to significantly improve the overall survival, but in the subgroup with complete resection, chemotherapy did not make an apparent difference [6]. Additional consideration of chemotherapy is given in children younger than 2 years for whom RT is preferably delayed [7]. Neoadjuvant chemotherapy has been recommended as it improves the margin of tumor resection with a decrease in the incidence of intraoperative blood loss in children [11]. The best chemotherapy regimen is yet to be determined, but a combination utilizing platinum and etoposide as backbone is preferred [7].

Radiation therapy (RT) is an important aspect of management, but its implementation is limited in young children (< 2 years). Among a database of 524 patients, 5-year survival was better in irradiated CPC patients [13]. A systematic review has also shown better 5-year overall survival among patients who received radiation therapy in comparison with those who did not receive RT (47.4 ± 6.5% versus 25.2 ± 4.3%) [6]. In another meta-analysis, Mazloom *et al*. [14] support the use of RT as it improved the progression-free survival as well as the overall survival [14]. In addition, they also established that patients receiving complete craniospinal irradiation had a significantly increased progression-free survival as well as overall survival than those who received limited RT treatment volumes such as whole-brain or involved-field RT [14]. However, the decision of craniospinal irradiation will have to be made on a case-by-case basis after a complete MRI evaluation of the spine and examination of cerebrospinal fluid (CSF). Our patient has also received adjuvant RT, as he belonged to a higher age group.

Despite CPC carrying a poor prognosis, our patient underwent a successful gross total resection (GTR) and received postoperative radiotherapy. He made a satisfactory recovery, with a further plan to review with gadolinium-enhanced neuroimaging at a later date.

## Conclusion

We conclude that, when possible, GTR should be a priority in the management of patients with CPC. However, in the event of subtotal resection, neoadjuvant chemoradiotherapy may be given for a second surgery to achieve a complete GTR. Adjuvant radiotherapy and chemotherapy have been shown to improve survival in a recent meta-analysis. However, more studies need to be carried out for a global consensus.

## Data Availability

The datasets used and/or analyzed during the current study are available from the corresponding author on reasonable request.
